# GDF-15 as a biomarker for diagnosis and prognosis of lung cancer: a meta-analysis

**DOI:** 10.3389/fonc.2025.1447990

**Published:** 2025-03-12

**Authors:** Teng Pan, Rui Duan, Zihan Xu, Xiaohan He, Xiaojin Luo, Guanglin Zhou, Yu Song, Jinhai Deng, Xuerui Tan, Fengxiang Wei

**Affiliations:** ^1^ Institute of Medicine, Longgang District Maternity & Child Healthcare Hospital of Shenzhen City (Longgang Maternity and Child Institute of Shantou University Medical College), Shenzhen, China; ^2^ Doctor of Department of Pathology, The Ninth Medical Center of the Chinese PLA General Hospital, Beijing, China; ^3^ College of Biological Sciences, China Agricultural University, Beijing, China; ^4^ Department of Oncology, Cancer Institute, University College London, London, United Kingdom; ^5^ The Genetics Laboratory, Longgang District Maternity and Child Healthcare Hospital of Shenzhen City, Shenzhen, Guangdong, China; ^6^ Department of Otorhinolaryngology Head and Neck Surgery, Peking University 3rd Hospital, Beijing, China; ^7^ Richard Dimbleby Laboratory of Cancer Research, School of Cancer & Pharmaceutical Sciences, King’s College London, London, United Kingdom; ^8^ Clinical Research Center, First Affiliated Hospital of Shantou University Medical College, Shantou, Guangdong, China; ^9^ Institute of Medicine, Longgang Maternity and Child Institute of Shantou University Medical College, Shenzhen, China

**Keywords:** GDF-15, lung cancer, biomarkers, diagnostic utility, prognostic value, meta-analysis

## Abstract

**Introduction:**

Due to the tendency of lung cancer to be diagnosed at advanced stages, many patients are not eligible for curative surgery. Identifying early detection and prognosis biomarkers is crucial for improving outcomes. This study explores the potential of Growth Differentiation Factor 15 (GDF-15) as a biomarker for these purposes.

**Methods:**

A thorough review and meta-analysis of literature from PubMed, Embase, the CENTRAL, and the CNKI was performed. We analyzed the diagnostic accuracy of GDF-15, focusing on its sensitivity, specificity, and AUC. Additionally, we investigated the association between three-year overall survival and GDF-15 levels in lung cancer patients. Our analysis included nine studies, encompassing 1296 patients with lung cancer and 1182 healthy controls.

**Results:**

GDF-15 showed high diagnostic performance with a sensitivity of 0.80 (95% Confidence Interval (CI): 0.71-0.87), specificity of 0.92 (95% CI: 0.85-0.96), diagnostic odds ratio of 45 (95% CI: 25-79), and an AUC of 0.93 (95% CI: 0.90-0.95). Moreover, the prognosis analysis revealed that the plasma GDF-15 levels were significantly higher in patients than controls (standardized mean difference: 2.91, CI 2.79-3.04 and P < 0.00001), and the odds ratio of 3-year overall survival rate was 4.05 (95% CI: 1.92-8.51 and P = 0.0002).

**Discussion:**

GDF-15 exhibits strong potential as both a diagnostic and prognostic biomarker in lung cancer, distinguishing effectively between patients and healthy individuals. These findings support its further exploration and potential integration into clinical practice.

**Systematic Review Registration:**

https://www.crd.york.ac.uk/PROSPERO/, identifier CRD42024519807.

## Introduction

Lung cancer is the most common cancer worldwide and the leading cause of cancer-related deaths (1). Small cell lung cancer (SCLC) and non-small cell lung cancer (NSCLC) are the two main subtypes, accounting for 15% and 85% of all lung cancers, respectively (2). As a result of late detection and diagnosis, lung cancer has an overall high mortality rate (3). Predictive biomarkers have been well integrated into lung cancer management, and recent years have seen the emergence of liquid biopsy options. To predict and monitor patients’ response to tyrosine kinase inhibitor (TKi) therapy, plasma samples of lung cancer patients are tested for the EGFR p. (Tyr790Met) mutation (4). Additionally, there are also ongoing efforts aimed at improving clinically relevant biomarker detection to improve lung cancer outcomes (5). Lung cancer patients’ survival rates can be improved with liquid biopsies due to reduced laboratory turnaround times, expedited treatment initiation, and reduced laboratory costs (6). Numerous efforts are underway to develop and test the practicality of predictive, diagnostic, and prognostic biomarkers for lung cancer (7).

Growth differentiation factor 15 (GDF15) is a cytokine released during stress, and it is a unique member of the transforming growth factor-β (TGF-β) superfamily (8). Human GDF15 is encoded by a simple gene comprising two exons located on chromosome 19p12-13.1, with 309 bp exon I, 891 bp exon II and a single 1820 bp intron (9). Activated GDF15 or mature GDF15 is produced through several steps from unprocessed translated form of GDF15, which is 308 amino acids long including the signal sequence (29 aa), the propeptide (167 aa) and a mature protein (112 aa) (9). Accumulating studies revealed multifunctional roles of GDF15 in controlling biological events. GDF15 exerts its physiological effects through interactions with various cell surface receptors. Among these, the transforming growth factor-beta (TGF-β) receptor, GDNF-family receptor a-like (GFRAL), and the CD48 receptor are of particular interest. For instance, binding of GDF15 to GFRAL controls food intake and body mass, and helps to enhance of hippocampal neural stem cell proliferation and neuronal differentiation. Furthermore, GDF15 binds to TGF-β receptors, leading to the phosphorylation of SMAD2/3 and SMAD1/5/8, which promotes the progression and cancer cells (10). Additionally, the binding between GDF15 and CD48 accumulates FOXP3 in Tregs, enhancing its tumor-suppressing functions (11, 12). Collectively, these studies indicate the important role of GDF15 in maintaining systemic functions.

GDF15 has been well studied in different stages of cancer. Evidence has shown that GDF15 has been proved to enhance tumor cell proliferation (13). Also, GDF15 plays a pivotal role in metastasis. In colorectal cancer (CRC) patients, circulating GDF15 concentration has been observed to be elevated, and further increase when metastasis occurs (14, 15), suggesting GDF15 is positively associated with metastasis of CRC. Similarly, GDF15 facilitates metastasis of breast cancer cells to bone tissue, which can be blocked by inhibition of the receptor activator of nuclear factor-κB ligand (RANKL) (16). Also, higher levels of serum GDF15 are also detected in colorectal cancer patients and positively correlate with the occurrence of liver metastasis (17). Therefore, GDF15 is closely involved in the progression of cancer. Notably, in large-scale screenings, GDF15 has been identified as the most significantly over-expressed soluble factor and its concentration is correlated with the progression in cancer patients across different cancer types, especially NSCLC (18). Thus, its role in predicting disease progression or treatment outcomes of NSCLC needs to be further investigated.

In this study, we systemically analyzed all available literature regarding GDF15 as a biomarker for the diagnosis and prognosis of lung cancer.

## Methods

### Study registration

Eligible studies included studies evaluating GDF-15’s diagnostic or prognostic capabilities in lung cancer, particularly those quantifying sensitivity, specificity, and area under the receiver operating characteristic curve (AUC), as well as overall survival (OS). Studies excluded were review articles, basic science research, animal studies, letters, conference abstracts, studies with inaccessible data, studies with a high bias risk, and articles not in English or Chinese. The control group comprised healthy individuals. Primary Outcome were the measures of diagnostic accuracy. Secondary Outcomes were the plasma GDF-15 levels and three-year OS. This systematic review was registered with PROSPERO (CRD42024519807). Data from private patients isn’t collected in this systematic review; therefore, ethical approval isn’t needed.

### Search strategy

Teng Pan searched the Cochrane Central Register of Controlled Trials (CENTRAL), Medline (via PubMed), Embase, China National Knowledge Infrastructure (CNKI), Chinese Biomedical Literature Database (CBM), Chinese Scientific Journal Database (VIP), and Wan-Fang Database from their inception to January 24, 2024, without restrictions on publication status. **Table 1** shows the search strategy, designed in accordance with the Cochrane Handbook, adapted as needed for each database and repeated prior to final analysis to incorporate the latest studies.

**Table 1:** The search strategy.

Order	Strategy
*1	Search “Growth differentiation factor -15” OR “GDF-15” OR “Macrophage inhibitory factor-1” OR “MIC-1”
*2	Search: “diagnostic” OR “prognostic”
*3	Search: “cancer” OR “lung cancer” OR “SCLC” OR “NSCLC”
*4	Search: “humans” [MeSH Terms] NOT “animals” [MeSH Terms]
*5	*1 + 2*
*6	*2+*3 OR *1+*3
*7	*2+*3+*4
*8	1*+*2+*3+*4

### Study selection

This review spanned from January 24, 2023, to June 10, 2024. Reviewers were trained to understand the study’s aims and background thoroughly. Titles, abstracts, and keywords were independently screened by Teng Pan and Zihan Xu, including those from additional sources. And then identify potentially relevant studies, review their full texts, and document their exclusions. Xiaohan He and Xiaojin Luo arbitrated if consensus cannot be reached between the two reviewers.

### Data extraction and management

A standardized data extraction form was collaboratively developed. Data were extracted independently by Teng Pan and Zihan Xu, concerning general information, participant characteristics, methods, outcomes, and other relevant information. Xiaohan He and Xiaojin Luo discussed or arbitrated individual disagreements.

### Risk of bias assessment

Bias risk was assessed by Teng Pan and Zihan Xu using tools recommended by the Cochrane Collaboration. Based on the risk of bias, each category was rated low, high, or unclear.

### Data analysis and synthesis

Data was analyzed and synthesized using Review Manager (RevMan) version 5.3 and Stata version 15. Generally, continuous variables are analyzed using standardized mean differences with 95% confidence intervals (CIs), and dichotomous outcomes was analyzed using 95% CIs of risk ratios. Diagnostic utility of plasma GDF-15 levels was quantified by sensitivity, specificity, diagnostic odds ratio (DOR), AUC, positive likelihood ratio and the negative likelihood ratio, with their 95% CIs. Deek’s funnel plot and asymmetry test was used to assess publication bias. A random-effects model was applied when heterogeneity is low (I² < 50%), and a fixed-effects model when high (I² ≥ 50%) in the meta-analysis.

## Results

### Literature retrieval and screening

**Figure 1:**
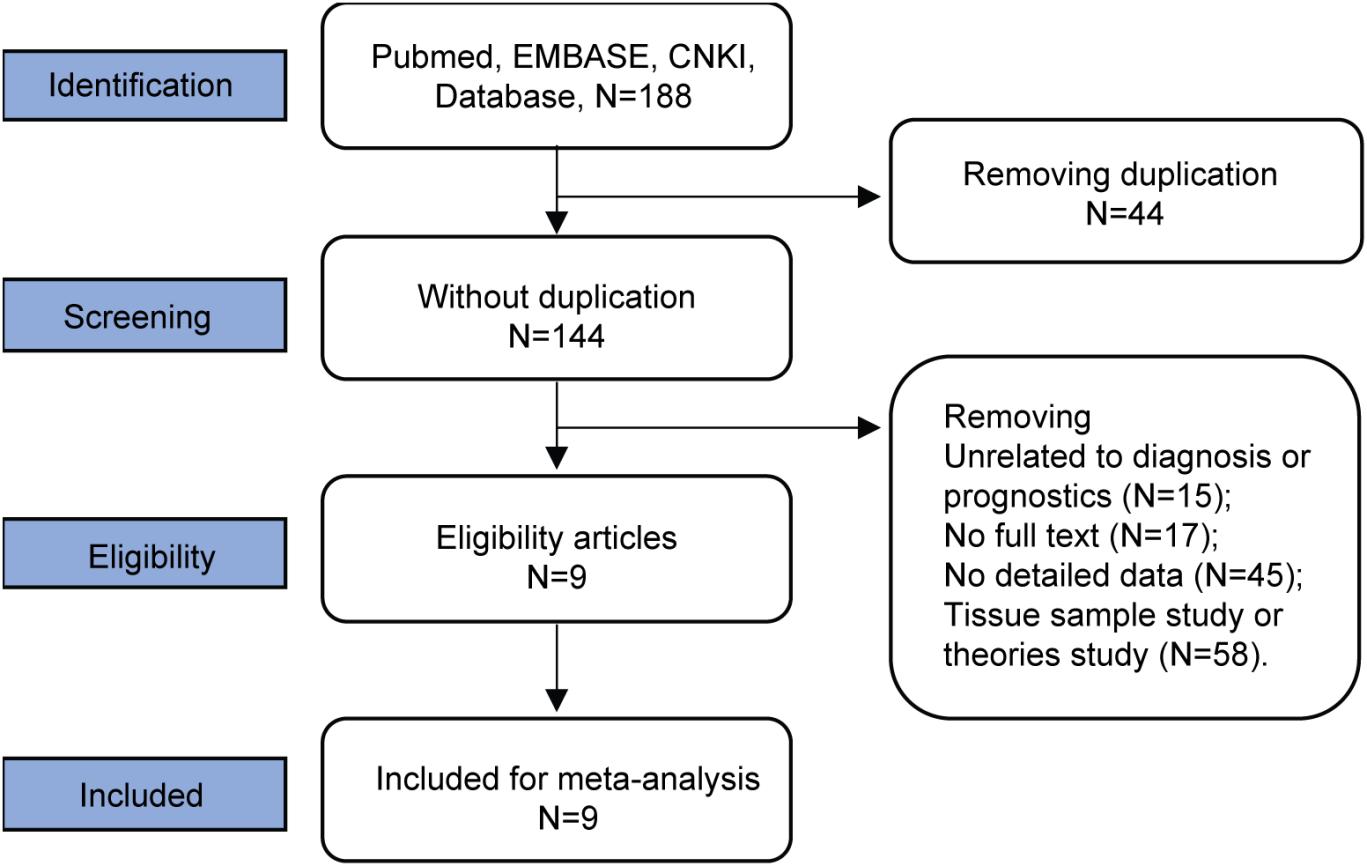
Flow chart of literature selection.

We conducted a systematic literature search that identified 188 articles, sourced from PubMed (32), Embase (25), and the China National Knowledge Infrastructure (CNKI) database (131). Our initial review resulted in the removal of 44 duplicate articles. Further exclusions included 17 articles due to unavailable full texts, 45 articles that lacked adequate data, 58 articles focused on tissue samples or theoretical research, and 15 articles unrelated to diagnostic or prognostic outcomes. Ultimately, only 9 articles met our criteria for inclusion for further analysis. The selected studies spanned the years 2011 to 2023 and comprised a cohort of 1,296 patients diagnosed with lung cancer alongside 1,182 healthy controls, all located in China. The article screening process is provided in **Figure 1**. Final included studies are summarized in **Table 2**. **Table 3** delineates the basic characteristics and the OS metrics among the patient cohort.

**Table 2:** Basic characteristics of included articles.

Study ID	Sample Size (P/C)	Cutoff pg/ml	AUC	Sensitivity	Specificity	Survival outcome
Liu 2016 (19)	152/105	1000	0.9	70.4%	99%	3-year OS
Xu 2020 (20)	296/240	1000	0.906	63.50%	95.0%,	–
Deng 2021 (21)	88/41	1199.05	0.851	78.4%	71.00%	–
Zhao 2022 (20)	126/126	1013.21	0.821	76.2%	89%	3-year OS
Fu 2011 (22)	79/200	1000	0. 916	64. 6%	97%	–
Yang 2022 (23)	93/70	1000	0.758	89.8%	88.6%	3-year OS
Li 2023 (24)	70/70	1165.79	0.823	90%	80%	–
lv 2019 (25)	72/50	–	–	–	–	–
Pei 2019 (26)	320/280	1000	–	84. 2%	90. 0%	–

P/C: lung cancer patients/health control.

**Table 3:** Basic characteristics of the 3-year OS in lung cancer patients.

Study ID	Sample Size (H/L)	Cutoff pg/ml	High-level subgroup 3-year OS	Low-level subgroup 3-year OS
Liu 2016 (19)	58/94	1465	77.6%	94.8%
Yang 2022 (23)	43/50	1000	65.12%	86%

H/L, High-level GDF-15; L, Low-level.

### Quality evaluation of the included studies

**Figure 2:**
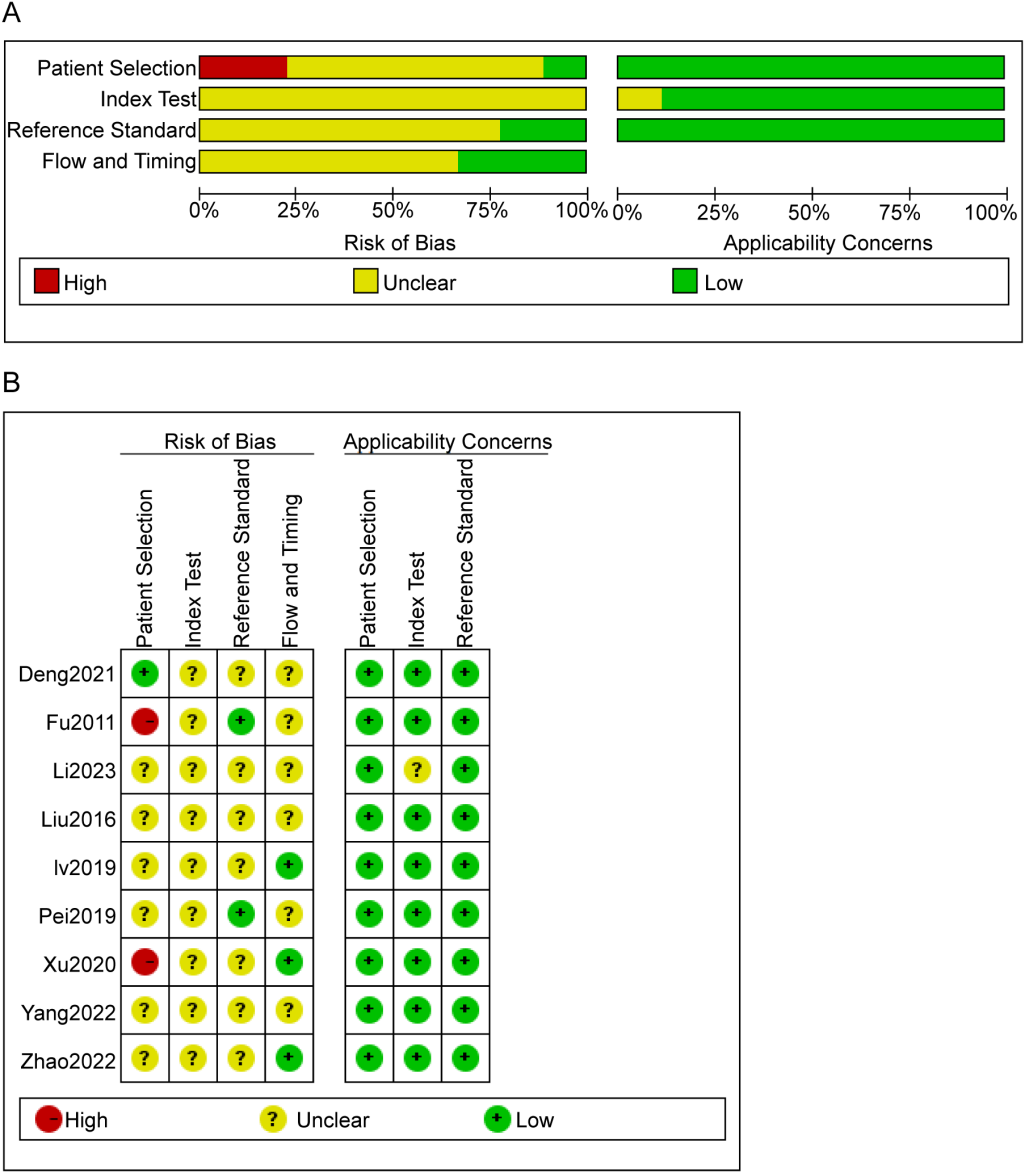
Risk of bias evaluation results. **(A)** Risk of bias summary. The quality assessment of each literature was shown. The color red, yellow and green indicate high, unclear and low risk of bias, respectively. **(B)** Risk of Bias Graph. Literature quality was evaluated based on four criteria. Each criterion has three bias assessment ratings—high, unclear, and low—represented by the colors red, yellow, and green, respectively.

Within the nine studies analyzed, two articles (20, 22)failed to adequately delineate their inclusion and exclusion criteria. Additionally, three critical methodological aspects were uniformly ambiguous across all studies: the enrollment strategy, whether participants were randomized or consecutively enrolled, the application of blinding in assessing index test outcomes, and the timing between conducting the index test and the reference standard. Despite these issues, there was no indication of incomplete data, selective reporting, or other biases in the studies, as illustrated in **Figure 2**.

### Diagnostic meta-analysis

**Figure 3:**
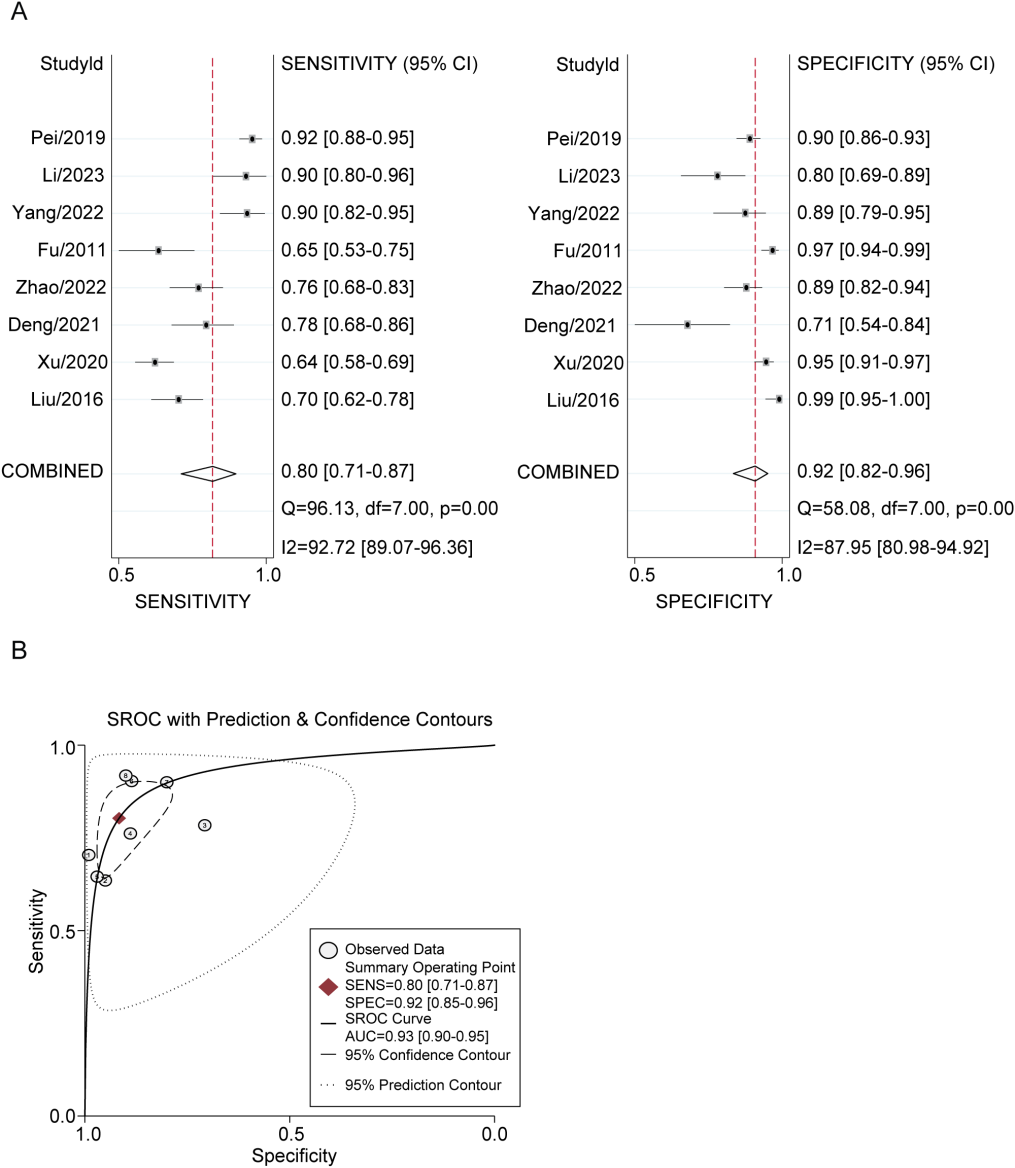
Plasma GDF-15 Levels for Lung Cancer Diagnosis. **(A, B)** Forest Plots of Overall Pooled Data for GDF-15: **(A)** sensitivity and specificity used to diagnose lung cancer. **(B)** AUC used to diagnose lung cancer.

The diagnostic utility of plasma GDF-15 levels was quantified using STATA 15.0. The meta-analysis revealed a pooled sensitivity of 0.80 (95% CI: 0.71-0.87) and a specificity of 0.92 (95% CI: 0.85-0.96) for GDF-15 in distinguishing lung cancer patients from healthy individuals. The DOR was 45 (95% CI: 25-79) and the AUC was 0.93 (95% CI: 0.90-0.95). The positive likelihood ratio was 9.6 (95% CI: 5.5-16.9), and the negative likelihood ratio was 0.22 (95% CI: 0.15-0.31). Significant heterogeneity was present, with I^2^ values of 92.72% (p < 0.001) for sensitivity and 87.95% (p < 0.001) for specificity (**Figure 3**).

### Sensitivity analysis of publication bias

**Figure 4:**
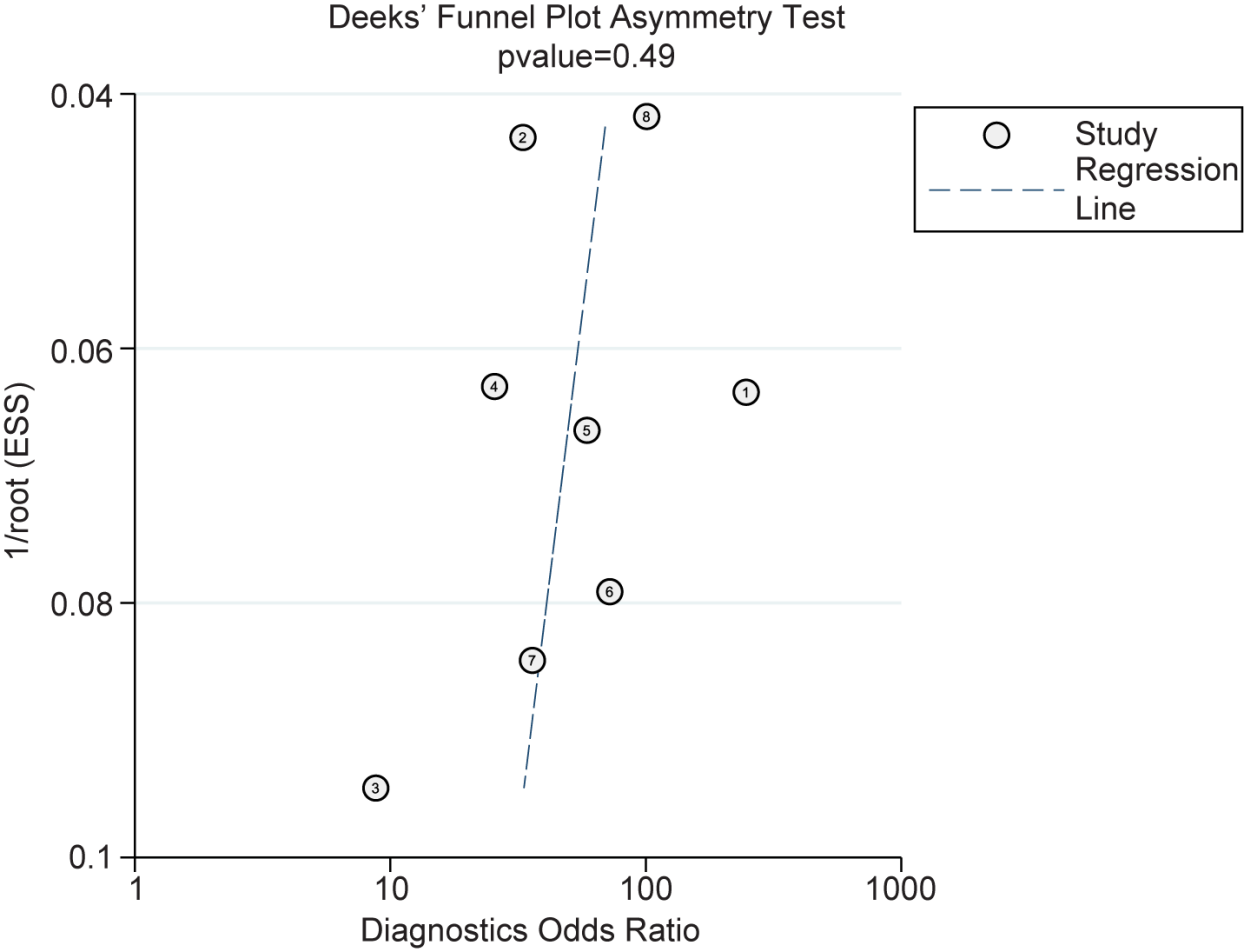
The Deeks’ Funnel Plot Asymmetry for Publication Bias.

Then we evaluated the publication bias of DOR. The asymmetry test showed P = 0.49, suggesting no significant publication bias among the studies included in this study (**Figure 4**).

### Plasma GDF-15 levels

**Figure 5:**
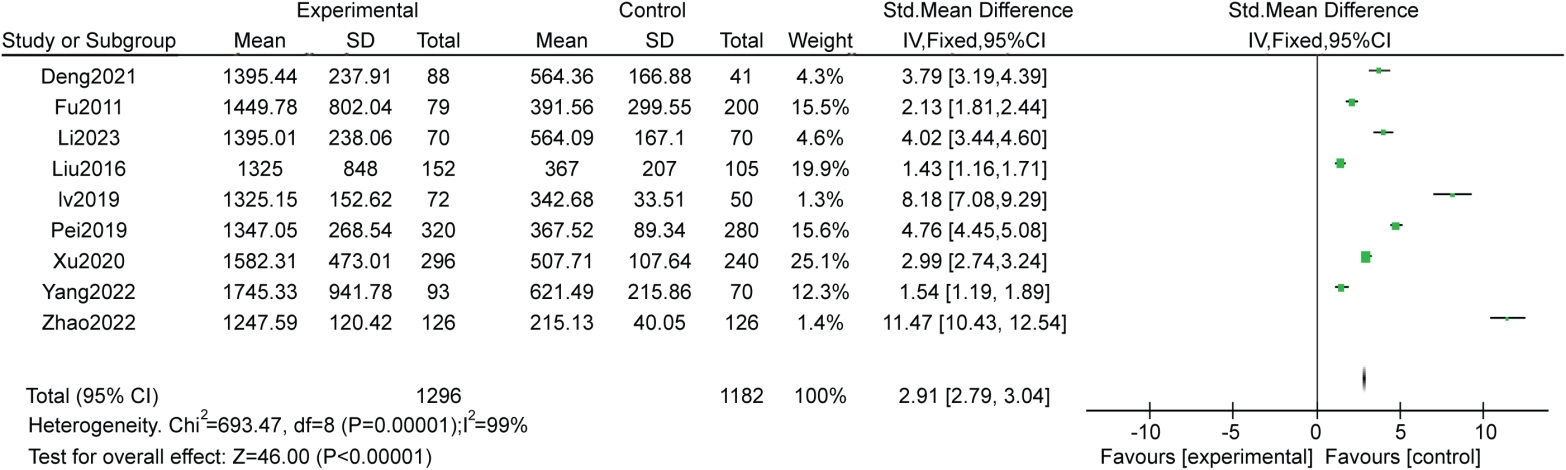
Forest plots of the overall pooled data for plasma GDF-15 level.

We assessed and compared plasma GDF-15 levels between lung cancer patients and healthy controls by meta-analysis. Due to the extremely high heterogeneity (I² = 99%, P < 0.00001), a fixed-effect model was employed for analysis. The overall standardized mean difference was 2.91, with a 95% CI 2.79-3.04 and P < 0.00001. Hence, the plasma GDF-15 levels showed statistical significance between lung cancer patients and healthy controls in the meta-analysis (**Figure 5**).

### Prognostic significance and 3-year overall survival

**Figure 6:**
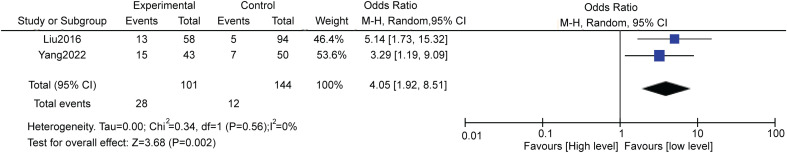
Forest plots of the overall pooled data for 3-year overall survival in GDF-15 subgroups.

Meta-analysis of 3-year overall survival rate revealed significant differences between patients with high and low levels of GDF-15. The odds ratio was 4.05 (95% CI: 1.92-8.51), indicating a robust association between elevated GDF-15 levels and decreased survival (P = 0.0002). Heterogeneity for this analysis was nonexistent (I² = 0%, P = 0.56) (**Figure 6**).

## Discussion

Previous studies have established the close involvement of GDF15 in different cancer stages. Although GDF15 upregulation is observed in various cancers, its roles, whether pro-tumorigenic or anti-tumorigenic, can differ based on the cancer type and stage (27). A significant association of elevated GDF15 levels with poor cancer prognosis is suggestive of its pro-tumorigenic nature. GDF15 has been intricately involved in several stages of oncogenesis, including tumor initiation, proliferation, metastasis, drug resistance, and recurrence. In breast cancer, GDF15 overexpression drives the epithelial-mesenchymal transition (EMT) phenotype through the IGF-1R-FoxM1 signaling pathway (28). Furthermore, GDF15 is able to facilitate metastasis of breast cancer cells to bone tissue via the activation of the receptor activator of nuclear factor-κB ligand (RANKL) (29). Collectively, these studies proved the pro-tumorigenic effects.

In our study, we focused on the role of GDF15 in lung cancer. Multiple studies have previously found that GDF-15 is abnormally highly expressed in NSCLC tissues and patient serum (21, 30, 31). A prospective cohort study observed that higher baseline levels of GDF-15 in patients with type 2 diabetes were associated with a higher future risk of tumors, including lung cancer (32). Also, another prospective study also found that elevated GDF-15 levels in the elderly population were related to an increased lung cancer risk (33). These studies were evidenced by another clinical finding, which emphasized that GDF15 expression is positively correlated with progression and chemotherapy resistance in lung cancer cohorts (21). Collectively, high levels of GDF-15 are positively linked to poor prognosis in patients with NSCLC, manifesting as shorter overall survival (34), higher risk of lymph node metastasis (21), and higher recurrence rates (30). Correspondingly, in this study, our findings corroborate the diagnostic utility of GDF-15 in NSCLC, evidenced by its high sensitivity (80%), specificity (92%), and an AUC of 0.93. GDF15 levels were notably higher in lung cancer patients than in healthy controls. Thus, our study has enhanced the diagnostic role of GDF15 in NSCLC.

Mechanistically, the functions of GDF15 are exerted via both tumor cell-intrinsic and -extrinsic signalings. Intrinsically, GDF-15 has been proved to promote proliferation, invasion, migration, but decrease apoptosis of NSCLC by activating PTEN/PI3K/AKT signaling pathway (35). Extrinsically, proinflammatory cytokines, such as TNFα, IL1β, and IL6, have been found to induce GDF15 expression (36). In addition, NF-κB, a pivotal pro-inflammatory regulator, can augment GDF15 expression by binding exon 2 of *GDF15* gene, thereby promoting its transcription (37). Collectively, these findings underscore the intricate relationship between inflammation and GDF15. Considering that inflammation is very common previously and concomitantly to tumor development, GDF15 is supposed to be involved in the initiation and progression of tumor. Especially, GDF15 is involved in the regulation of different immune cells. Elevated GDF15 levels are associated with diminished lymphocyte infiltration into tumors, suggesting its potential role in mediating immune evasion (38). Notably, GDF15 was found to suppress the expression of key immune factors, including INF-γ, t-bet, TNF-α, Granzyme B, and perforin (39). Tumor-derived GDF15 has been shown to attenuate the cytotoxicity of macrophages, thereby hindering macrophage-mediated tumor surveillance during tumorigenesis (37). Furthermore, GDF-15 has been shown to inhibit T cell infiltration into the tumor microenvironment, potentially reducing the efficacy of immunotherapy (40).

Notably, like other members of the TGFβ family, although the role of GDF15 in promoting tumorigenesis has been highlighted in numerous studies, solid evidence has documented that GDF15 also has anti-tumorigenic effects. GDF15 has demonstrated capabilities in inhibiting cellular proliferation, amplifying apoptosis rates, and suppressing tumor growth (40). For instance, in glioblastoma studies, GDF15 overexpression was shown to impede tumor growth and reduce tumor volume in immunocompromised mouse models (41). Similar inhibitory effects of GDF15 were observed in colorectal and bladder cancers (42). Some studies have found that increased expression of GDF-15 significantly inhibited the proliferation of NSCLC cells (31), suggesting that it may have a tumor-suppressive effect. On a molecular mechanism level, one study found that EZH2 suppresses GDF-15 expression by binding to the GDF-15 promoter region and inducing H3K27 trimethylation, which may be related to the poor prognosis of NSCLC (31). Taken together, it can not be discarded that GDF-15 may also have a protective effect.

Since GDF15 is soluble, secreted factor, and can be detected in body fluids, especially circulation, thus making it as a dream detectable target in liquid biopsy, like serum. Previous meta-analyses have linked elevated GDF-15 levels in body fluids with poor prognosis across various solid tumors, showing a negative correlation with overall survival (17). Our results align with these findings, presenting an odds ratio of 4.05, indicating significantly worse outcomes for patients with high GDF-15 levels. Therefore, our study further proved the development of detection kits targeting GDF15 for the diagnosis and prognosis prediction of patients with NSCLC. It should be emphasized that in a latest phase 1-2a clinical trial study (GDFATHER-1/2a trial, NCT04725474), neutralizing GDF15 antibody has been developed and shown great performance in overcoming anti-PD-1 and anti-PD-L1 resistance in solid tumors, especially NSCLC (43), indicating that GDF15 is not only a diagnostic biomarker, but also an efficient treatment target. Of course, a further assessment of their safety and efficacy requires further clinical studies.

While there are some limitations to our analysis, it provides valuable insight into GDF-15’s role as a lung cancer diagnostic and prognostic marker. Further studies with larger, more diverse populations are necessary to validate our findings and elucidate GDF-15’s role in lung cancer pathogenesis. Additionally, longitudinal studies tracking GDF-15 levels over time in lung cancer patients could deepen our understanding of its prognostic significance. At last, significant heterogeneity attributed primarily to population homogeneity regarding country and ethnicity, may introduce bias and limit the generalizability of our findings. Moreover, the scant data on the association between three-year overall survival and GDF-15 levels could contribute to this heterogeneity and potential bias in prognostic analysis.

In conclusion, our analysis indicates that serum GDF-15 levels are significantly elevated in lung cancer patients compared to healthy individuals, serving as a useful biomarker for lung cancer diagnosis. Furthermore, elevated serum GDF-15 levels are associated with a poorer prognosis in lung cancer patients, underscoring their potential as a significant risk factor in clinical outcomes.

## Data Availability

The original contributions presented in the study are included in the article/supplementary material. Further inquiries can be directed to the corresponding authors.
